# To donate or not to donate? Future healthcare professionals’ opinions on biobanking of human biological material for research purposes

**DOI:** 10.1186/s12910-023-00930-z

**Published:** 2023-07-22

**Authors:** Jan Domaradzki, Justyna Czekajewska, Dariusz Walkowiak

**Affiliations:** 1 Department of Social Sciences and Humanities, Poznan University of Medical Sciences, Rokietnicka 7, St., Poznań, 60-806 Poland; 2 Department of Organization and Management in Health Care, Poznan University of Medical Sciences, Poznań, Poland

**Keywords:** Biobanks, Biobanking, Biomedical research, Donation, Future healthcare professionals, Medical students, Tissues, Ethical, legal and social issues

## Abstract

**Background:**

Over the last few decades biobanks have been recognised as institutions that may revolutionise biomedical research and the development of personalised medicine. Poland, however, still lacks clear regulations regarding the running of biobanks and the conducting of biomedical research. While the awareness of the general public regarding biobanks is low, healthcare professions and medical students also lack basic knowledge regarding biobanks, and such ignorance may affect their support for biobanks.

**Methods:**

This study is aimed at assessing the knowledge and attitudes of future healthcare professionals towards the donation of human biological material for research purposes and is based on a sample of 865 Polish medical students at Poznań University of Medical Sciences.

**Results:**

This research has shown that the awareness of medical students’ regarding biobanks is low. It has also shown that while the majority of future healthcare professionals enrolled in this study supported the idea of biobank research and declared themselves willing to donate, still many students felt ambivalent about the biobanking of human biological material for research purposes and expressed concerns over biobanking research. While the primarily motivation to participate in biobank research was the desire to help advance science and to develop innovative therapies, the most common reason for a refusal was the fear that the government, insurance companies or employers, might have access to the samples. Concerns over unethical use of samples and data safety were also prevalent. More than half of students opted for a study-specific model of consent and only a few opted for broad consent.

**Conclusions:**

This research suggests that a lack of knowledge about biobanks, their role and activities may affect medical students’ support for biobanks and their active participation in the collection and management of biospecimens for research purposes. Since in the future medical, nursing and pharmacy students will be involved in the collection, storage, testing and analysis of biospecimens from their patients, medical students in all professional fields should be trained regarding the concept, purposes and operational procedures of biobanks, as well as the ethical, legal and social implications of biobank research.

**Supplementary Information:**

The online version contains supplementary material available at 10.1186/s12910-023-00930-z.

## Background

Biobanks may be defined as a large collection of human biospecimens and associated personal and health information (health records, family history, lifestyle, genetic information) organised systematically predominantly for health and medical research [1, 2]. The first biobanks were established in the late 1990s as ‘population biobanks’ or ‘nationally delimited, population-based genetic databases’ and it is estimated that in Europe alone there are currently more than 500 biobanks that store approximately 100 million samples of human biological material (HBM) [3].

Although the idea of biobanking is not new since HBM has been collected and stored in numerous European countries for over a century, over the last few decades there has been a huge progress in technical possibilities of generating, processing and linking the data. In fact, such technological advances as genetic engineering, the sequencing of the human genome, computerization, Artificial Intelligence (AI)-driven process automation, robotisation and the internet have revolutionized the management of biobanks. Consequently, biobanks have grown rapidly and have been recognised as institutions that may revolutionise biomedical research and become an important instrument for the advancement of science and the development of personalised medicine [4–9]. In 2009 *Time* magazine therefore included biobanks as one of the “10 ideas changing the world right now” [10]. Similarly, in 2019 *Forbes* magazine defined biobanking as a key area for “changing the world” [11]. This should come as no surprise because biobanks have become platforms for biomedical research and their potential for broadening scientific knowledge regarding genetic, behavioural and environmental determinants of many diseases, supporting the development of new therapies and diagnostic methods, as well as improving patients’ medical care, is widely recognised. Biobanks are also useful for analysis of the risks of behaviour such as smoking, alcohol abuse and drug use or diet [12]. Finally, they help to explain the way genetic factors tend to affect various psychiatric disorders [13, 14].

Simultaneously, while biobanks play a crucial role in the development of biomedical research, they also help to develop and implement national and international standards for procedures and management [15]. One of the first international organizations aiming at the harmonization of scientific, technical, ethical and legal issues related to biobanking was the International Society for Biological and Environmental Repositories (ISBER) founded in 2000, which provides a forum for communication between biobanks and creates opportunities for networking through annual meetings, working groups and discussions, and organizes education and training campaigns on both technical and ethical issues related to biotechnology [15–17]. Additionally, in 2013 the European Biobanking and BioMolecular resources Research Infrastructure (BBMRI)-European Research Infrastructure Consortium (ERIC) was launched. While the BBMRI-ERIC, which is one of the largest European research infrastructures, seeks to develop pan-European cooperation of biobanks, it also intends to develop standards and guidelines regarding ethical, legal and social issues related to biobanking [3, 7, 18].

At the same time, in order to raise awareness of the biobank industry and its global potential among the public, policymakers and research community numerous international and national scientific initiatives have been organized across Europe, including the European Biotech Week, which aim to educate the public about biotechnology, exchange good practices and improve engagement of various social actors in the field of biotechnology [19, 20]. Additionally, since collecting large number of biosamples and clinical data for research from large number of patients is essential for the success of biomedical research, biobanks have stared a cooperation with various patient organizations by launching educational campaigns, promotion of biobank research, organisation of meetings and round-tables and incorporating patients representatives in biobank’s advisory boards [21, 22].

Finally, since the future of biobanking requires involvement of medial students, medical and health experts biobanks have also started offering the new education courses, specialized trainings and university and postgraduate programs on the latest advances in biobanking biotechniques and international standards for biobank research regarding data protection and sharing [17, 23, 24].

Despite all efforts aimed at ensuring the development of biobanking, there are still difficulties that the scientific community has to face. Especially, that while the Polish Biobanking Network (Polska Sieć Biobanków – PSB) was established in 2016 within the BBMRI-ERIC [25–28] and since then more than 50 biobanks joined the PSB, Poland still lacks clear regulations for biobanking and conducting biomedical research [29]. While studies suggest that Poles tend to feel positive about biobanks, support the idea of creating such institutions in Poland and declare a willingness to donate^1^ their biospecimens for research purposes [30–32], the knowledge of general public on biobanks and biomedical research is sparce. Similar to other countries, many Poles have, in fact, never heard the term ‘biobank’, confuse participation in biomedical research with medical examinations and lack even basic knowledge regarding biobanking [30, 33].

This is important because the success of any biobank depends on building social trust toward biomedical research and research institutions that might help establish co-operation with local communities and guarantee the constant participation of a large number of donors who will donate their biospecimens for research purposes [34, 35]. At the same time, biobanks also require constant co-operation with healthcare professionals (HCPs) who may help to persuade patients to donate their biospecimens left over after medical procedures [36–41]. Research also suggests that HPCs, including physicians and nurses, lack a basic knowledge of biobanks and that such ignorance may affect their attitudes towards donation for research purposes and support for biobanks [37, 39, 42–44]. Future HCPs also reveal gaps in their knowledge regarding biobanks [45–48].

While it is crucial to raise the public’s awareness about biobanking, it is equally important to educate HCPs about biobanks and engage physicians, nurses and pharmacists in promoting the donation biospecimens for biomedical research. Not only are they a reliable source of information on biomedical research and explain the implications of patient’s genetics, but they also help dispel moral and ethical doubts related to such donations for research purposes [15, 36–38, 40, 41]. This study aims to assess knowledge and attitudes of medical students towards the donation of HBM for research purposes.

## Methods

The data were collected between December 2021 and February 2022 among students enrolled at the Poznań University of Medical Sciences (PUMS) in Poland using an anonymous self-administered online questionnaire on medical students’ attitudes toward the donation of HBM for biobank research.

The questionnaire used for the purpose of this study was a self-developed questionnaire elaborated according to the guidelines of the European Statistical System [49] and constructed from themes based on a review of the literature [33, 50–53] and the study aim. It contained 19 open-ended questions designed to explore the key issues relating to research biobanks and were divided into four domains. The first assessed students’ knowledge on research biobanks (whether they have heard about research biobanks, their impressions related to the word *biobank*, and awareness of what biobank is). The second domain included questions regarding students’ attitudes towards participation in biobank research (the willingness to participate in biobank research, motivation to donate and refuse biosamples for research purposes). The third domain referred to ethical and legal issues related to biobanking of human biological material (control over data sharing, preferred type of consent, protecting the rights and identity of the donors, and for withdrawing consent, as well as issues related to the ownership and profit sharing). The last section of the questionnaire included questions concerning students’ demographic characteristics, including gender, faculty and year of study (Supplementary material).

Participants were included if they were enrolled in PUMS and were keen to participate in the study. An invitation to participate in the study was posted on an online platform. A total of 865 students responded and completed the survey.

The final version of the questionnaire was posted on an online platform and distributed among medical, nursing and pharmacy students via a communication platform used at PUMS for educational purposes during the COVID-19 pandemic. Students received an invitation letter and were informed of the study’s purpose, as well as the voluntary, anonymous and confidential character of the study. Participants completed self-administered, computer-assisted questionnaires using electronic devices. The questionnaires took approximately 20 min to complete and were collected anonymously.

This study was performed in line with the principles of the Declaration of Helsinki [54]. After ethics approval and research governance approval were obtained from the PUMS Bioethics Committee (KB – 926/21) all students received a letter of invitation to participate in the study and informed consent was obtained from all respondents who agreed to take part in the study.

The data collected in the questionnaires were verified and checked for completeness, quality and consistency, and were exported into the statistical packages JASP (Version 0.16.3.0) and PQStat v.1.8.4. The results are presented as descriptive statistics. Pearson’s Chi-square and the Fisher’s exact test were used where it was appropriate to assess the differences in the distribution of answers among the groups. Kruskal–Wallis’ ANOVA was applied to compare the answers to five Likert-scale questions, after which Dunn’s post-hoc analysis with Bonferroni's correction was used to determine statistically significant differences between the three groups of students. A 5% level of significance was used for all the hypothesis tests.

## Results

A total of 865 medical students participated in the study (Table 1). The sample consisted of 673 women (77.8%) and 192 men (22.2%), all of Polish origin. While the majority were enrolled in their first or second year of study (30.5% and 23.2% respectively), medical students (MS) predominated (48.1%) over nursing students (NS) (27.5%) and pharmacy students (PS) (24.4%).

**Table 1:** Socio-demographic characteristics of students

	1. Medical students (MS) n (%)	2. Nursing students (NS) n (%)	3. Pharmacy students (PS) n (%)
**Gender**			
Female	281 (67.5)	226 (95)	166 (78.7)
Male	135 (32.5)	12 (5)	45 (21.3)
**Year of the study**			
1	99 (23.8)	86 (36.1)	79 (37.5)
2	99 (23.8)	54 (22.7)	48 (22.7)
3	81 (19.5)	40 (16.8)	27 (12.8)
4	56 (13.5)	29 (12.2)	30 (14.2)
5	43 (10.3)	29 (12.2)	27 (12.8)
6	38 (9.1)	-	-

Although the vast majority of students stated that they had heard of biobanks, NS’ awareness of biobanks was statistically significantly lower than those of MS’ and PS’ (NS: 61.8% vs MS: 76% and PS: 73.9%) (Table 2). While almost half the students from each group felt positive about biobanks (PS: 53.1%, MS: 48.6%, NS: 46.6%), many respondents had mixed feelings related about them (MS: 27.9%, PS: 26.5%, NS: 24.8%). The majority of students associated biobanks with either scientific or research-related institution (MS: 54.6%, PS: 49.8%, NS: 44.5%) or an institution related to medicine or health (NS: 52.1%, PS:48.8%, MS: 43%), and only 21 students from all three faculties (2.4%) did not associate them with such institutions.

**Table 2:** Medical students’ knowledge regarding biobanks

	1. Medical students (MS) n (%)	2. Nursing students (NS) n (%)	3. Pharmacy students (PS) n (%)	*p* for differences between groups
**Have your ever heard about biobanks?**				
Yes	316 (76)	147 (61.8)	156 (73.9)	**1 vs. 2 *p* < 0.0001**
No	100 (24)	91 (38.2)	55 (26.1)	**2 vs. 3 *p* < 0.0001**
**What are your impressions when you hear the word *biobank*?**				
Positive	202 (48.6)	111 (46.6)	112 (53.1)	ns
Negative	3 (0.7)	3 (1.3)	2 (1)	
Mixed, both positive and negative	95 (22.8)	65 (27.3)	41 (19.4)	
I do not know. It’s irrelevant to me	116 (27.9)	59 (24.8)	56 (26.5)	
**Do you think biobanks are institutions:**				
Financial or money related	5 (1.2)	4 (1.7)	2 (0.9)	ns
Scientific or research-related	227 (54.6)	106 (44.5)	105 (49.8)	
Related to medicine or health	179 (43)	124 (52.1)	103 (48.8)	
Related to the police and/or the military	1 (0.2)	0 (0)	0 (0)	
I don’t know	4 (1)	4 (1.7)	1 (0.5)	

The statistically significant results are given in boldface

Almost three quarters of students (73.3%) declared a willingness to donate their HBM for research purposes (MS: 74.5%, PS: 74.4%, NS: 70.2%) (Table 3). At the same time, students indicated many possible reasons for refusal. While the most common reason for a refusal to donate was the fear that the government (62.8% in total; MS: 65%, PS: 65%, NS: 57.1%), insurance companies (62.7%; MS: 66.8%, PS: 54% and NS: 47.9%) or employers (53.3%; MS: 61.5%, PS: 51.7%, NS: 40.3%), might have access to the samples, MS were more concerned about this than NS and PS. Students were also concerned over possible unethical use of the samples, the safety of their data (PS: 57.8%, MS: 57.9% and NS: 51.7%), and the possibility of commercial use of the samples (PS: 65.4%, NS: 63.8% and MS: 60.4%). Additionally, almost half the students from each group would by discouraged by the necessity to travel (MS: 58.8%, PS: 52.1% and NS: 48.8%). At the same time, while PS were more afraid of the invasive nature of the sampling procedure (29.9% vs NS: 14.3% and MS: 16.1%), MS were the least afraid of the risk of being infected with HIV (28.4% vs NS: 44.2% and PS: 38.2%).

**Table 3:** Medical students’ attitudes towards donation for biobank research

	Definitely not n (%)	Rather not n(%)	I do not know n (%)	Rather yes n (%)	Definitely yes n (%)	Median (IQR)	*P* for groups differences
**If you were asked, would you donate a sample of your biological material to a biobank for research purposes?**							
1. Medical students (MS)	8 (1.9)	36 (8.7)	62 (14.9)	214 (51.4)	96 (23.1)	4 (3–4)	ns
2. Nursing students (NS)	10 (4.2)	17 (7.1)	44 (18.5)	119 (50)	48 (20.2)	4 (3–4)	
3. Pharmacy students (PS)	2 (0.9)	16 (7.6)	36 (17.1)	124 (58.8)	33 (15.6)	4 (3–4)	
**What are the reasons for your refusal to donate to biobank?**							
Physical distance and the necessity to travel							
MS	14 (3.4)	131 (31.5)	26 (6.3)	192 (46.1)	53 (12.7)	4 (2–4)	ns
NS	11 (4.6)	86 (36.1)	25 (10.5)	88 (37)	28 (11.8)	3 (2–4)	
PS	4 (1.9)	85 (40.3)	12 (5.7)	84 (39.8)	26 (12.3)	4 (2–4)	
The necessity to repeat examination							
MS	12 (2.9)	185 (44.5)	26 (6.2)	156 (37.5)	37 (8.9)	3 (2–4)	ns
NS	11 (4.6)	115 (48.3)	15 (6.3)	77 (32.4)	20 (8.4)	2 (2–4)	
PS	10 (4.7)	91 (43.1)	11 (5.2)	78 (37)	21 (10)	3 (2–4)	
Fear over the safety of the data							
MS	30 (7.2)	130 (31.3)	15 (3.6)	132 (31.7)	109 (26.2)	4 (2–5)	ns
NS	12 (5)	97 (40.8)	6 (2.5)	73 (30.7)	50 (21)	4 (2–4)	
PS	20 (9.5)	63 (29.9)	6 (2.8)	80 (37.9)	42 (19.9)	4 (2–4)	
Fear over unethical use of the sample							
MS	33 (7.9)	111 (26.7)	11 (2.7)	112 (26.9)	149 (35.8)	4 (2–5)	ns
NS	10 (4.2)	51 (21.4)	6 (2.5)	74 (31.1)	97 (40.8)	4 (2–5)	
PS	13 (6.2)	48 (22.7)	4 (1.9)	65 (30.8)	81 (38.4)	4 (2–5)	
Fear over the invasive nature of the sampling procedure (pain, sight of blood, needles or injections)							
MS	170 (40.9)	170 (40.9)	9 (2.1)	47 (11.3)	20 (4.8)	2 (1–2)	**1 vs. 3 *p* < 0.0001**
NS	90 (37.8)	110 (46.2)	4 (1.7)	25 (10.5)	9 (3.8)	2 (1–2)	**2 vs. 3 *p* < 0.0001**
PS	60 (28.4)	83 (39.3)	5 (2.4)	38 (18)	25 (11.9)	2 (1–4)	
Fear of being infected with HIV							
MS	108 (25.9)	183 (44)	7 (1.7)	57 (13.7)	61 (14.7)	2 (1–4)	**1 vs. 2 *p* < 0.0001**
NS	33 (13.9)	85 (35.7)	11 (4.6)	53 (22.3)	56 (23.5)	3 (2–4)	**1 vs. 3 *p* < 0.001**
PS	39 (18.5)	74 (35.1)	11 (5.2)	37 (17.5)	50 (20.7)	2 (2–4)	
Fear over detection of disease or genetic predispositions							
MS	195 (46.9)	174 (41.8)	16 (3.8)	26 (6.3)	5 (1.2)	2 (1–2)	ns
NS	115 (48.3)	101 (42.5)	5 (2.1)	15 (6.3)	2 (0.8)	2 (1–2)	
PS	99 (46.9)	86 (40.8)	2 (0.9)	17 (8.1)	7 (3.3)	2 (1–2)	
Fear that the data generated from the research might result in stigmatisation and discrimination							
MS	100 (24)	183 (44)	12 (2.9)	85 (20.4)	36 (8.7)	2 (2–4)	ns
NS	59 (24.8)	104 (43.7)	11 (4.6)	42 (17.6)	22 (9.3)	2 (2–4)	
PS	48 (22.7)	100 (47.4)	9 (4.3)	31 (14.7)	23 (10.9)	2 (2–4)	
Fear over the commercial use of the samples							
MS	21 (5)	123 (29.6)	21 (5)	153 (36.8)	98 (23.6)	4 (2–4)	ns
NS	13 (5.5)	60 (25.2)	13 (5.5)	86 (36.1)	66 (27.7)	4 (2–5)	
PS	19 (9)	42 (19.9)	12 (5.7)	88 (41.7)	50 (23.7)	4 (2–4)	
Fear that the government might have access to the samples							
MS	25 (6)	100 (24)	21 (5)	143 (34.4)	127 (30.6)	4 (2–5)	ns
NS	18 (7.6)	65 (27.3)	19 (8)	70 (29.4)	66 (27.7)	4 (2–5)	
PS	11 (5.2)	54 (25.6)	9 (4.2)	78 (37)	59 (28)	4 (2–5)	
Fear that insurance companies might have the access to the samples							
MS	18 (4.3)	99 (23.8)	21 (5.1)	132 (31.7)	146 (35.1)	4 (2–5)	**1 vs. 2*p* < 0.0001**
NS	20 (8.4)	73 (30.7)	31 (13)	62 (26.1)	52 (21.8)	3 (2–4)	**1 vs. 3** ***p* < 0.01**
PS	16 (7.6)	63 (29.9)	18 (8.5)	64 (30.3)	50 (23.7)	4 (2–4)	
Fear that employers might have the access to the samples							
MS	30 (7.2)	110 (26.5)	20 (4.8)	132 (31.7)	124 (29.8)	4 (2–5)	**1 vs. 2*p* < 0.0001**
NS	33 (13.9)	88 (37)	21 (8.8)	50 (21)	46 (19.3)	2 (2–4)	**1 vs. 3** ***p* < 0.05**
PS	22 (10.4)	60 (28.4)	20 (9.5)	65 (30.8)	44 (20.9)	4 (2–4)	

The statistically significant results are given in boldface

70.9% of all students declared that they would donate their HBM for biobank research because they believed that it would benefit science, generating new knowledge and developing therapies for many diseases (MS: 74.1%, PS: 72% and NS: 70%) (Table 4). 11.1% also wanted to know their health status (MS: 9.4%, NS: 13.7% and PS: 12.6%).

While more than half the students (51.9%) declared that they would opt for a study-specific model of consent (PS: 54.5%, NS: 53.8% and MS: 49.5%), 16.6% opted for blanket consent (MS: %, NS: % and PS: %), and only a few opted for broad consent (NS: 10.5%, PS: 10% and MS: 9.6%).

Although the vast majority of students in every group believed that donors’ biosamples should be reversibly coded (89.8%, PS: 93.4%, NS: 90.3% and MS: 87.7%,) rather than anonymized (7.4%, MS: 8.7%, NS: 7.1% and PS: 5.2%), MS believed that biosamples could be included in an opt-in method, while NS supported an opt-out method of consent. Finally, while many students said that, when donors want to withdraw from the research, their biosamples should be anonymized, MS tended to say that they should be destroyed.

**Table 4:** Medical students’ motivations to donate for biobank research

	1. Medical students n (%)	2. Nursing students n (%)	3. Pharmacy students n (%)	*P* for groups differences
**What would be your primary motivation for donating your biological material to a biobank?**				
To benefit society and future generations	28 (6.9)	12 (5.1)	12 (5.8)	ns
To advance science, help in generating new knowledge and develop therapies for various diseases	301 (74.1)	163 (70)	149 (72)	
To benefit my family, relatives and myself	32 (7.9)	21 (9)	18 (8.7)	
To receive medical treatment/service	2 (0.5)	2 (0.9)	0 (0)	
To know my health status	38 (9.4)	32 (13.7)	26 (12.6)	
To receive financial gratification	5 (1.2)	3 (1.3)	2 (0.9)	
**What type of consent would you prefer when donating your samples to a biobank?**				
Blanket (open-ended permission without any limitations and the need to renewed consent)	77 (18.5)	40 (16.8)	27 (12.8)	ns
Specific consent (for one experiment with well-defined aim / before every research that involves my samples)	206 (49.5)	128 (53.8)	115 (54.5)	
Broad consent (general consent for a broad range of future studies but subjected to specified limitations and restrictions stated in the consent form)	40 (9.6)	25 (10.5)	21 (10)	
Tiered consent (individually selected categories of research or research uses e.g. specific diseases, i.e. cancer or neurological diseases, or research conducted only by specified institutions, i.e. publicly-funded but not private)	35 (8.4)	17 (7.1)	26 (12.3)	
Consent delegated to bioethical committee	50 (12)	23 (9.7)	18 (8.5)	
I don’t know	8 (2)	5 (2.1)	4 (1.9)	
**Samples taken from donors for research purposes should be**				
Pseudonymized (reversibly coding, i.e. in case of detecting a disease)	365 (87.7)	215 (90.3)	197 (93.4)	ns
Anonymized (irreversibly coded, so that donor data cannot be determined)	36 (8.7)	17 (7.1)	11 (5.2)	
I don’t know	15 (3.6)	6 (2.5)	3 (1.4)	
**While donation the samples the donors should rather**				
Specify the types of research for which their specimens may be used	168 (40.4)	123 (51.7)	84 (39.8)	**1 vs. 2 *p* < 0.01**
Specify the types of research for which their specimens may not be used	209 (50.2)	90 (37.8)	95 (45)	**2 vs. 3 *p* < 0.05**
I don’t know	39 (9.4)	25 (10.5)	32 (15.2)	
**When donors want to withdraw from research their samples should be**				
Anonymized (irreversible coded) but available for further research	87 (20.9)	43 (18.1)	51 (24.2)	**1 vs. 3 *p* < 0.01**
Destroyed	237 (57)	131 (55)	93 (44.1)	
Prohibited from use in further research	72 (17.3)	53 (22.3)	57 (27)	
I don’t know	20 (4.8)	11 (4.6)	10 (4.7)	

The statistically significant results are given in boldface

When asked about the type of information they would like to receive before donating their HBM to a biobank, nearly all students were primarily interested in knowing the type and purpose of the study they would be donating to (MS: 99.6%, PS: 99.5% and NS: 99.2%) and who would have access to the research results (NS: 98.3%, MS: 97.6% and PS: 95.7%) (Table 5). At the same time, some statistically significant differences were found in students’ opinions on the control over data sharing. MS wanted to know who would own the biobank and how long their biosamples would generally be stored more often than NS and PS. PS were less interested in receiving information about the place where donors’ samples would be stored and the conditions for withdrawing their samples and data from the biobank than NS.

**Table 5:** Medical students’ opinions on control over data sharing

	Definitely not n (%)	Rather not n (%)	I do not know n (%)	Rather yes n (%)	Definitely yes n (%)	Median (IQR)	*P* for groups differences
**What information would you like to receive before submitting samples to the biobank?**							
Type and purpose of the research							
1. Medical students (MS)	1 (0.2)	1 (0.2)	0 (0)	27 (6.5)	387 (93.1)	5 (5–5)	ns
2. Nursing students (NS)	0 (0)	1 (0.4)	1 (0.4)	7 (3)	229 (96.2)	5 (5–5)	
3. Pharmacy students (PS)	0 (0)	0 (0)	1 (0.5)	8 (3.8)	202 (95.7)	5 (5–5)	
Who conducts research and where							
MS	0 (0)	18 (4.3)	5 (1.2)	87 (20.9)	306(73.6)	5 (4–5)	ns
NS	0 (0)	5 (2.1)	4 (1.7)	55 (23.1)	174(73.1)	5 (4–5)	
PS	2 (1)	12 (5.7)	3 (1.4)	52 (24.6)	142(67.3)	5 (4–5)	
Who owns the biobank							
MS	3 (0.7)	37 (8.9)	20 (4.8)	120 (28.8)	236 (56.7)	5 (4–5)	**1 vs. 2 *p* < 0.05**
NS	3 (1.3)	28 (11.8)	16 (6.7)	80 (33.6)	111 (46.6)	4 (4–5)	**1 vs. 3 *p* < 0.0001**
PS	3 (1.4)	43 (20.4)	12 (5.7)	64 (30.3)	89 (42.2)	4 (3–5)	
How long samples will be stored							
MS	6 (1.4)	25 (6)	14 (3.4)	104 (25)	267 (64.2)	5 (4–5)	**1 vs. 3 *p* < 0.01**
NS	3 (1.3)	18 (7.6)	7 (2.9)	55 (23.1)	155 (65.1)	5 (4–5)	**2 vs. 3 *p* < 0.01**
PS	5 (2.4)	22 (10.4)	7 (3.3)	70 (33.2)	107 (50.7)	5 (4–5)	
Where the samples will be stored							
MS	3 (0.7)	51 (12.3)	16 (3.9)	120 (28.8)	226 (54.3)	5 (4–5)	**2 vs. 3 *p* < 0.05**
NS	3 (1.3)	20 (8.4)	11 (4.6)	71 (29.8)	133 (55.9)	5 (4–5)	
PS	7 (3.3)	26 (12.3)	8(3.8)	76 (36)	94 (44.6)	4 (4–5)	
Who will have access to the results							
MS	1 (0.2)	9 (2.2)	0 (0)	67 (16.1)	339 (81.5)	5 (5–5)	ns
NS	0 (0)	3 (1.3)	1 (0.4)	34 (14.3)	200 (84)	5 (5–5)	
PS	1 (0.5)	8 (3.8)	0 (0)	42 (19.9)	160 (75.8)	5 (5–5)	
Conditions for withdrawing samples and data from the biobank							
MS	0 (0)	11 (2.6)	8 (1.9)	69 (16.6)	328 (78.9)	5 (5–5)	**2 vs. 3 *p* < 0.05**
NS	1 (0.4)	5 (2.1)	3 (1.3)	32 (13.4)	197 (82.8)	5 (5–5)	
PS	3 (1.4)	5 (2.4)	5 (2.4)	44 (20.8)	154 (73)	5 (4–5)	
Penalties for investigators who commit abuses							
MS	4 (0.9)	24 (5.8)	13 (3.1)	101 (24.3)	274 (65.9)	5 (4–5)	ns
NS	0 (0)	5 (2.1)	8 (3.4)	53 (22.3)	172 (72.3)	5 (4–5)	
PS	1 (0.5)	11 (5.2)	8 (3.8)	35 (16.6)	156 (73.9)	5 (4–5)	

The statistically significant results are given in boldface

Students were also asked what constitutes the most sensitive data that should be protected (Table 6). While most students from each group believed that all personal information should be protected (MS: 65.6%, PS: 60.6%, NS: 57.6%), it was national identification number (PESEL) (MS: 98.9%, NS: 97.1%, PS: 97.1%) and address (PS: 99.1%, MS: 98.6%, NS: 97.9%) that were considered as the most important data that should be protected. However, many students also indicated the information about their health status (PS: 66.3%, NS: 65.5%, MS: 63.7%), including information about their genetic susceptibility to somatic diseases (MS: 61.8%, PS: 57.8%, NS: 55%) and mental disorders (MS: 64.7%, PS: 61.2%, NS: 57.6%), addictions (MS: 56.7%, PS: 56.4%, NS: 48.8%) and diseases that run in the family (PS: 63%, MS: 62.1%, NS: 57.5%). Surprisingly, although some of the information listed are not collected by Polish biobanks, many students were also anxious about the protection of other personal information, although they differed in their opinions as to whether information on profession, nationality/ethnic group, political preferences, religion/confession and sex life should be protected.

**Table 6:** Medical students’ opinions on the type of information about the donor that should be protected

	Definitely not n (%)	Rather not n (%)	I do not know n (%)	Rather yes n (%)	Definitely yes n (%)	Median (IQR)	p for groups differences
**Which information about the donor should be protected?**							
Address data							
1. Medical students (MS)	1 (0.2)	5 (1.2)	0 (0)	49 (11.8)	361 (86.8)	5 (5–5)	ns
2. Nursing students (NS)	2 (0.8)	2 (0.8)	1 (0.4)	25 (10.5)	208 (87.4)	5 (5–5)	
3. Pharmacy students (PS)	0 (0)	0 (0)	2 (0.9)	21 (10)	188 (89.1)	5 (5–5)	
The national identification / identity number							
MS	1 (0.2)	3 (0.7)	1 (0.2)	31 (7.5)	380 (91.4)	5 (5–5)	ns
NS	1 (0.4)	5 (2.1)	1 (0.4)	20 (8.4)	211 (88.7)	5 (5–5)	
PS	1 (0.5)	4 (1.9)	1 (0.5)	8 (3.8)	197 (93.3)	5 (5–5)	
Health condition/previous diseases							
MS	49 (11.8)	97 (23.3)	4 (1)	53 (12.7)	213 (51)	5 (2–5)	ns
NS	23 (9.7)	57 (24)	2 (0.8)	41 (17.2)	115 (48.3)	4 (2–5)	
PS	21 (10)	46 (21.8)	4 (1.9)	44 (20.8)	96( 45.5)	4 (2–5)	
About addictions							
MS	60 (14.4)	116 (27.9)	4 (1)	64 (15.4)	172 (41.3)	4 (2–5)	ns
NS	31 (13)	85 (35.7)	6 (2.5)	38 (16)	78 (32.8)	3 (2–5)	
PS	26 (12.3)	63 (29.9)	3 (1.4)	37 (17.5)	82 (38.9)	4 (2–5)	
About diseases in the family							
MS	60 (14.4)	92 (22.1)	6 (1.4)	63 (15.2)	195 (46.9)	4 (2–5)	ns
NS	32 (13.4)	68 (28.6)	1 (0.4)	52 (21.8)	85 (35.7)	4 (2–5)	
PS	22 (10.4)	55 (26.1)	1 (0.5)	47 (22.3)	86 (40.7)	4 (2–5)	
About genetic susceptibility to somatic diseases							
MS	57 (13.7)	96 (23.1)	6 (1.4)	59 (14.2)	198 (47.6)	4 (2–5)	ns
NS	30 (12.6)	72 (30.3)	5 (2.1)	47 (19.7)	84 (35.3)	4 (2–5)	
PS	22 (10.4)	62 (29.4)	5 (2.4)	37 (17.5)	85( 40.3)	4 (2–5)	
About genetic susceptibility to mental disorders							
MS	50 (12)	92 (22.1)	5 (1.2)	64 (15.4)	205 (49.3)	4 (2–5)	ns
NS	28 (11.8)	67 (28.1)	6 (2.5)	48 (20.2)	89 (37.4)	4 (2–5)	
PS	19 (9)	61 (28.9)	2 (0.9)	40 (19)	89 (42.2)	4 (2–5)	
About sex life							
MS	27 (6.5)	78 (18.8)	7 (1.7)	85 (20.4)	219 (52.6)	5 (2–5)	ns
NS	14 (5.9)	48 (20.2)	8 (3.4)	57 (23.9)	111 (46.6)	4 (2–5)	
PS	11 (5.2)	35 (16.6)	10 (4.7)	52 (24.7)	103 (48.8)	4 (3–5)	
About religion/confession							
MS	25 (6)	56 (13.5)	24 (5.8)	80 (19.2)	231 (55.5)	5 (3–5)	**1 vs. 2 *p* < 0.05**
NS	21 (8.8)	53 (22.3)	13 (5)	42 (17.7)	110 (46.2)	4 (2–5)	**2 vs. 3 *p* < 0.05**
PS	9 (4.3)	26 (12.2)	12 (5.7)	50 (23.7)	114 (54)	5 (4–5)	
About profession							
MS	30 (7.2)	114 (27.4)	17 (4.1)	85 (20.4)	170 (40.9)	4 (2–5)	**1 vs. 2 *p* < 0.001**
NS	24 (10.1)	99 (41.6)	6 (2.5)	35 (14.7)	74 (31.1)	2 (2–5)	**2 vs. 3 *p* < 0.01**
PS	15 (7.1)	55 (26.1)	12 (5.7)	48 (22.7)	81 (38.4)	4 (2–5)	
About earnings							
MS	15 (3.6)	56 (23.5)	16 (3.8)	98 (23.6)	231 (55.5)	5 (4–5)	**1 vs. 2 *p* < 0.001**
NS	17 (7.1)	56 (23.5)	9 (3.8)	52 (21.9)	104 (43.7)	4 (2–5)	**2 vs. 3 *p* < 0.05**
PS	6 (2.8)	30 (14.2)	9 (4.3)	59 (28)	107 (50.7)	5 (4–5)	
About nationality / ethnic group							
MS	42 (10.1)	140 (33.7)	13 (3.1)	62 (14.9)	159 (38.2)	4 (2–5)	**2 vs. 3 *p* < 0.05**
NS	28 (11.8)	92 (38.6)	6 (2.5)	39 (16.4)	73 (30.7)	2 (2–5)	
PS	19 (9)	58 (27.5)	10 (4.8)	41 (19.4)	83 (39.3)	4 (2–5)	
About political preferences							
MS	18 (4.3)	43 (10.3)	24 (5.8)	83 (20)	248 (59.6)	5 (4–5)	**1 vs. 2 *p* < 0.001**
NS	16 (6.7)	42 (17.7)	19 (8)	51 (21.4)	110 (46.2)	4 (3–5)	**2 vs. 3 *p* < 0.01**
PS	10 (4.7)	24 (11.4)	9 (4.3)	44 (20.8)	124 (58.8)	5 (4–5)	
All information should be protected							
MS	38 (9.2)	82 (19.7)	23 (5.5)	85 (20.4)	188 (45.2)	4 (2–5)	ns
NS	27 (11.3)	48 (20.2)	26 (10.9)	47 (19.8)	90 (37.8)	4 (2–5)	
PS	21 (10)	43 (20.4)	19 (9)	42 (19.9)	86 (40.7)	4 (2–5)	

The statistically significant results are given in boldface

While some inter-group differences were found in students’ opinions on when donors should be asked for permission to use their biosamples (Table 7), most respondents in every group declared that such permission should be obtained in cases where the biospecimens would be used by external researchers (NS: 94.1%, PS: 91.1% and MS: 89.9%) or foreign institutions (NS: 90.8%, PS: 88.7% and MS: 85.4%). However, many students also believed that such permission should be also obtained when a new research project differs from the original project (NS: 88.3%, PS: 88.2% and MS: 82%). Interestingly, while less than one third of students in every group declared that no additional permission is required if if the donor consent while donating (MS: 31.3%, PS: 27% and NS: 19%) the majority declared that it should be required before every new research (NS: 76.1%, PS: 75.9% and MS: 69.5%).

**Table 7:** Medical students’ opinions regarding when the donors should be re-contacted

		Definitely not n (%)	Rather not n (%)	I do not know n (%)		Rather yes n (%)	Definitely yes n (%)	Median (IQR)	*P* for groups differences
**When should the biobank ask donors for permission to use their samples?**									
Before every new research									
1. Medical students (MS)	27 (6.5)		80 (19.2)	20 (4.8)	119 (28.6)		170 (40.9)	4 (2–5)	**1 vs. 2 *p* < 0.01**
2. Nursing students (NS)	9 (3.8)		37 (15.5)	11 (4.6)	54 (22.7)		127 (53.4)	5 (4–5)	
3. Pharmacy students (PS)	7 (3.3)		34 (16.1)	10 (4.7)	62 (29.4)		98 (46.5)	4 (4–5)	
If a new research project differs from the original project									
MS	15 (3.6)		45 (10.8)	15 (3.6)	130 (31.3)		211 (50.7)	5 (4–5)	**1 vs. 2 *p* < 0.01**
NS	4 (1.7)		18 (7.6)	6 (2.5)	57 (24)		153 (64.3)	5 (4–5)	
PS	3 (1.4)		17 (8)	5 (2.4)	66 (31.3)		120 (56.9)	5 (4–5)	
If it is intended to be used by researchers outside the institution donors donated to									
MS	10 (2.4)		25 (6)	7 (1.7)	85 (20.4)		289 (69.5)	5 (4–5)	**1 vs. 2 *p* < 0.05**
NS	2 (0.8)		8 (3.4)	4 (1.7)	39 (16.4)		185 (77.7)	5 (5–5)	
PS	2 (0.9)		15 (7.1)	2 (0.9)	37 (17.6)		155 (73.5)	5 (4–5)	
If it is intended for use by foreign institutions									
MS	11 (2.6)		40 (9.6)	10 (2.4)	96 (23.1)		259 (62.3)	5 (4–5)	**1 vs. 2 *p* < 0.05**
NS	2 (0.8)		15 (6.3)	5 (2.1)	42 (17.7)		174 (73.1)	5 (4–5)	
PS	2 (0.9)		18 (8.6)	3 (1.4)	50 (23.7)		138 (65.4)	5 (4–5)	
Does not have to ask if the doner consent while donating									
MS	140 (33.6)		110 (26.4)	36 (8.7)	78 (18.8)		52 (12.5)	2 (1–4)	ns
NS	104 (43.7)		53 (22.3)	12 (5)	46 (19.3)		23 (9.7)	2 (1–4)	
PS	78 (37)		52 (24.6)	24 (11.4)	30 (14.2)		27 (12.8)	2 (1–4)	

The statistically significant results are given in boldface

While students tended to be uniform in their opinions as to whether donors should be paid for tissue bank participation (MS: 32.9%, PS: 30.3% and NS: 25.6%) (Table 8), MS declared that donors hold the right to the samples donated for research purposes more often than NS and PS (28.4%, 18.5% and NS: 19.4% respectively). NS were less likely than MS and PS to support biobanks profiting from the research (17.7%, 28.4% and 29.4% respectively).

**Table 8:** Medical students’ opinions regarding ownership and profit sharing

	1. Medical students n (%)	2. Nursing students n (%)	3. Pharmacy students n (%)	*P* for groups differences
**Do you think donors should receive financial compensation for donating samples?**				
Yes	137 (32.9)	61 (25.6)	64 (30.3)	ns
No	102 (24.5)	57 (24)	46 (21.8)	
I do not know	177 (42.6)	120 (50.4)	101 (47.9)	
**Who should own the rights to the samples donated to the biobank**				
Biobank	57 (13.7)	26 (10.9)	26 (12.3)	**1 vs. 2 *p* < 0.05**
Donors	118 (28.4)	46 (19.4)	39 (18.5)	**1 vs. 3 *p* < 0.05**
Both biobank and donor	221 (53.1)	150 (63)	138 (65.4)	
I do not know	20 (4.8)	16 (6.7)	8 (3.8)	
**Who should profit from the biobank research?**				
Sponsor of the research/Biobank owner	118 (28.4)	42 (17.7)	62 (29.4)	**1 vs. 2 *p* < 0.01**
Donors	3 (0.7)	6 (2.5)	6 (2.8)	**2 vs. 3 *p* < 0.05**
Both biobank and donor	205 (49.3)	136 (57.1)	99 (46.9)	
I don’t know	90 (21.6)	54 (22.7)	44 (20.9)	

The statistically significant results are given in boldface

## Discussion

Although Poland has been a part of the European biobanking research infrastructure for almost a decade [25–27] and many Polish biobanks are highly specialised units, many are yet to implement a system of quality control and lack specific guidelines on methods of quality assurance for biobanking processes [28] or a system of proper ethical, legal and social issues (ELSI) to ensure the safety and privacy of donors and their data [55–57]. At the same time, although new Quality Management System has been created recently [15, 57], still, there remain no legal regulations unifying the activities of biobanks and biomedical research in Poland. Biobank research therefore provokes a heated debate over such issues as informed consent, data protection and sharing, and profit-making, which might discourage possible donors [58–61]. For all these reasons, over the past few years medical experts and the government have debated the need to provide legislative rules to govern the correct operation of biobanks that would ensure suitable standards for obtaining, storing, and working with HBM [62]. Another factor that might limit the successful process of collecting and processing high-quality biospecimens for Polish biobanks is the lack of knowledge and support of (future) HCPs, who might assist biobanks in several aspects of their activities [36–41].

This research therefore supports the findings from other studies that showed that both medical students and HCPs possess limited knowledge regarding biobanks and feel reluctant to participate actively in biobank research. While the vast majority of students enrolled in this study said they had heard about biobanks, supported the idea of biobank research and declared themselves willing to donate, still many future HCPs felt ambivalent about the biobanking of HBM for research purposes and expressed concern over biobanking research. Similarly, studies conducted in other countries also showed that while medical students had heard about biobanks and generally felt positive about biobank research, the vast majority possessed no knowledge about biobanking, and were not aware of the existence of biobanks in their countries [45–47].

Thus, our research confirms that there is an urgent need to broaden MS’ knowledge about biobanks, since limited awareness of biobanks, their role and their activity was also found among practising HCPs. In fact, irrespectively from the country, previous studies demonstrated that HCPs in Australia, Colombia, Côte d’Ivoire, Morocco, Saud Arabia, Egypt, Pakistan either had never heard about biobanks and did not know the existence of biobanks in their countries, or did not understand properly their role and the purpose of biobanks, and were unfamiliar with biobanking Standard Operating Procedures (SOPs) [37, 39, 43, 44, 63–65].

Another important finding is that the majority of Polish students declared a willingness to donate their biosamples for research purposes (MS: 74.5%, NS: 70.2% and 74.4% PS). This is in line with observation made by other studies that (future) HCPs generally supported the use of biosamples for research purposes, and were willing to transfer their biological material and share personal data [39, 42, 48]. More importantly, medical students enrolled in this study were primarily motivated by the desire to help advance science and to develop innovative therapies (70.9%). Similar altruistic motivations were found in other countries, where both medical students and practicing physicians believed that donations would advance biomedical research and help to develop new diagnostic and therapeutic methods [44, 47, 48, 63, 64].

At the same time, most students expected detailed information about the biobank, the biomedical research they would donate to and data protection. However, this should not surprise since according to the General Data Protection Regulation (GDPR) that have been implemented in Poland by the Personal Data Protection Act of May 2018 all personal data have to be protected, and the Polish biobanks guidelines of good practices and data protection follow international and European regulations and recommendations created by international organizations regarding that matter (i.e. BBMRI-ERIC and ISBER) [15, 26, 57].

This research demonstrates that several important factors served as future HCPs’ rationale for non-participation. The majority of respondents enrolled in this study were anxious about the unethical or commercial misuse of their biosamples, mainly fearing that their private data might be accessible to insurance companies, the government and employers. However, such anxieties related to biobank donation were found also outside Europe where legislation regarding data protection is different. In fact, research shows that both medical students and HCPs in Australia, Egypt, Saudi Arabia or Colombia were also concerned about data confidentiality, possible misuse of their biosamples and discrimination or possible misuse of biospecimens for either unethical research, i.e. human cloning or commercial purposes [44, 47, 64–66].

This study also supports other finding that suggest that among (future) HCPs there is no agreement regarding a preferred type of consent. While most respondents believed that permission should be obtained from the donors every time their HBM is to be used for research, less than one third of students opted for blanket consent or broad consent. This should not surprise, since both MSs and HCPs in other countries raised questions about the broad consent model used in hospital biobank recruitment process and stressed the need for full control, governance and accreditation [37, 44, 67].

All in all, this research shows that, although medical students believe that biobanks represent a great opportunity for the advancement of personalised medicine and the development of innovative diagnostic and therapeutic methods, their awareness of biobanks is poor. More importantly, it suggests that, although most future HCPs declared themselves willing to donate and perceived the many benefits of biobanking, students’ lack of knowledge affects theirs attitudes towards biobank research that were often perceived as risky and ethically problematic [45–48]. Thus, while this study helps to understand factors that influence the attitudes of medical students towards biobanking, it also shows that in order to enhance their integration into a biobank research, knowledge about biobanking and its practices among future HCPs should be further developed. This is important, because while researchers and biobank administrators agree that there is a need to establish special awareness campaigns that would help to increase the awareness of a general public on biobanks and promote public participation in biobank research [68, 69], it is equally important to educate future HCPs, including nurses, pharmacists and physicians about biobank research, so that in the future they might engage in the promotion of biobanks and the collection of biospecimens from donors [36–44, 64–66]. This is in line with the suggestion that the future of biobanking in Poland and elsewhere requires the development of new education courses for both medical students and medical and health experts [17, 24].

Simultaneously, it is crucial to recognize that comprehensive education in biobanking should extend beyond physicians and nurses to encompass all relevant specialties necessary in biobanking. This research highlights the importance of educational programs that should cover the latest advancements in biotechnology, including genetic engineering, genome sequencing techniques, AI-driven process automation, data analytics, and robotics, but also incorporate a deep understanding of international and national ethical, legal, and societal standards [15, 57, 70]. By integrating these diverse elements into educational curricula, HCPs can assume a more proactive role in advocating for research biobank donations [6, 7]. Empowering HCPs with knowledge about ethical considerations, legal frameworks, and societal expectations equips them to engage with patients and the wider community, fostering trust and promoting the value of contributing to research biobanks [71]. By broadening the scope of education to encompass multidisciplinary perspectives, HCPs gain a comprehensive understanding of the intricacies surrounding biobanking. This enables them to effectively communicate the benefits of participation, address concerns, and navigate ethical dilemmas that may arise.

In conclusion, it is imperative to go beyond technical advancements and incorporate education on ethical, legal, and societal aspects to empower HCPs in their efforts to encourage and advocate for research biobank donations. By integrating these dimensions into educational programs, we can enhance the active engagement of HCPs and facilitate the promotion of donation for research biobanks.

## Limitations

Although this study has shed some light on the knowledge and attitudes of future HCPs in Poland towards the biobanking of HBM for research purposes, it has some limitations. Firstly, this research was conducted during the COVID-19 pandemic, which hindered the recruitment process and reduced the number of respondents. Secondly, this study covers responses from medical students enrolled at only one Polish medical university, which has a local dimension. For both of these reasons the results of this study cannot be extrapolated to cover the entire population of medical students either in Poznań or in Poland as a whole. Future studies should therefore include a larger group of MS from other Polish medical universities and compare them with those from other countries. Thirdly, this study is based solely on the quantitative method, so, in order to better understand future HCPs attitudes towards biobanking research, including their motivations, expectations and anxieties related to donation, further in-depth studies using qualitative methods are required. Fourthly, since only few studies on the attitudes of (future) healthcare professionals on the donation of HBM for research purposes were conducted in Europe, and the vast majority were conducted in countries that are not subject to the same EU legislation regarding data collecting, storing and protection, it can be difficult to make comparisons between these countries. Especially, that they also differ in the way culture influence people’s attitudes and trust towards science, biomedical research and donation for research purpose. Finally, we analyzed students’ opinions on donation and not their decisions to donate for the purpose of biomedical research, so it should be stressed that intentions and behaviors often differ.

Despite these limitations, however, there are benefits to this study that may stimulate further studies. Most importantly, because there is scarcity of research on the attitudes of medical students toward biobanking in Poland, it may help to stimulate the discussion on the role of (future) HCPs in promoting biomedical research and supporting the data collection process. By identifying the gaps in medical students’ knowledge regarding biobanks it may also help to identify factors and issues that might impede the successful integration of tissue donation and biobank research into routine hospital practices.

## Conclusion

This study has shown that gaps in knowledge regarding biobanks, their role and their activities might affect MSs’ support for biobanks and their active participation in the collection and management of biospecimens for research purposes. This is important because in the future medical, nursing and pharmacy students will work in various research institutions, hospitals, universities and private clinics or pharmaceutical companies, and will be involved in the collection, storage, testing and analysis of HBM. We therefore suggest that, in order to overcome this knowledge deficit, medical students in all professional fields should receive more education about the importance of biobanks and precision medicine. At the same time, while they should be trained about the concept, purposes, and operational procedures of biobanks, the future of biobanking also requires the development of new education courses, for both medical students and medical and health experts, that should include not only programs on the latest biotechnological advances, but also international and national ethical, legal and societal standards for biobank research, including informed consent, level of participation, data protection and sharing, the return of research results and the profit-making. Finally, since the biobanking in Poland is still in an early stage of development and there is currently no uniform regulatory system that applies to research biobanks, there is a urgent need to implement standards that would facilitate the functioning of biobanks in the county [15, 28, 29, 57]. Additionally, standardised guidelines for in all Europe countries are also recommended, that would facilitate the sharing of biosamples and information and cooperation among researchers across different countries and the protection of donors’ rights.

## Supplementary Information

**Additional file 1.**

## Data Availability

The datasets generated during the study are available from the corresponding author on reasonable request.

## Abbreviations

BBMRI-ERIC: Biobanking and BioMolecular resources Research Infrastructure-European Research Infrastructure Consortium
ELSI: Ethical, legal, and social issues
GDPR: General Data Protection Regulation
HBM: Human biological material
HCPs: Healthcare professionals
ISBER: International Society for Biological and Environmental Repositories
MS: Medical students
NS: Nursing students
PS: Pharmacy students
PSB: Polish Biobanking Network
PUMS: Poznań University of Medical Sciences
SOPs: Standard Operating Procedures

## Footnotes

1 While in this paper we use the word *donate* we are aware that some authors suggest that the word *share* might be more appropriate, since it does not imply the transfer of the title and possession, and stresses that the subject or patient who offers one’s biospecimens for research purposes remains its owner, and at any time has decisions about its use. We are grateful to the anonymous reviewer for bringing our attention to this.
