# Supplementary material for: To donate or not to donate? Future healthcare professionals’ opinions on biobanking of human biological material for research purposes
Source: BMC Med Ethics. 2023 Jul 22;24:53. doi: 10.1186/s12910-023-00930-z (PMC10363302; doi:10.1186/s12910-023-00930-z)
Supplement: Supplementary file 1 — Additional file 1. [file 12910_2023_930_MOESM1_ESM.pdf]

Dear Madam! Dear Sir!
My name is Jan Domaradzki and I work at Poznan University of Medical Sciences. Together with my research team we are doing a project to better understand medical students' knowledge and attitudes towards biobanking of human biological material for research purposes. We would like to invite you to share your opinion on this important topic.
This survey should only take up to 15 minutes of your time. At the same time, we assure that this survey is completely anonymous and confidential. As all responses to anonymous they cannot be traced back to the respondent. Moreover, while no personally identifiable information is captured your responses will be combined with those of many others and summarized in a report to further protect your anonymity. All information gathered will be used only for scientific purposes.
For any questions, or if you need assistance to complete this questionnaire, please contact:

Jan Domaradzki, dr hab. n. hum.
Laboratory of Health Sociology and Social Pathology, Department of Social Sciences and Humanities
Poznan University of Medical Sciences
Rokietnicka 7, Poznań, Poland
tel./fax: 61 8452 770; e-mail: jandomar@ump.edu.pl

Your feedback is very important. We appreciate your time and help

Sincerely

Jan Domaradzki

----

1. Gender
☐ Woman
☐ Man

2. Faculty
☐ Medicine
☐ Nursing
☐ Pharmacy

3. Year of study
☐ 1
☐ 2
☐ 3
☐ 4
☐ 5
☐ 6

4. Have your ever heard about biobanks?
☐ Yes
☐ No

5. What are your impressions when you hear a word *biobank*?
☐ Positive
☐ Negative
☐ Mixed, both positive and negative
☐ I do not know, it is irrelevant to me

6. Do you think biobanks are institutions:
☐ Financial or money related
☐ Scientific or research-related
☐ Related to medicine or health
☐ Related to the police and/or the military
☐ I do not know

7. If you were asked, would you donate the sample of your biological material to a biobank for research purposes?

- Definitely yes
- Rather yes
- Rather no
- Definitely no
- I do not know

8. What would be your primary motivation for donating your biological material to a biobank?

- To benefit society and future generations
- To advance science, help in generating new knowledge and develop therapies for various diseases
- To benefit my family, relatives and myself
- To receive medical treatment/service
- To know my health status
- To receive financial gratification

9. Do you think donors should receive financial compensation for donating samples?

- Yes
- No
- I do not know

10. What information would you like to receive before submitting samples to the biobank?

	Definitely yes	Rather yes	Rather no	Definitely no	I do not know
Type and the purpose of the research					
Who conducts the research and where					
Who owns the biobank					
How long samples will be stored					
Where the samples will be stored					
Who will have access to the results					
Conditions for withdrawing samples and data from the biobank					
Penalties for investigators who commit abuses					

11. What are the reasons for your refusal to donate to biobank?

	Definitely yes	Rather yes	Rather no	Definitely no	I do not know
Physical distance and the necessity to travel					
The necessity to repeat examination					
Fear over the safety of the data					
Fear over unethical use of the sample					
Fear over the invasive nature of the sampling procedure (pain, sight of blood, needles and injections)					
Fear of being infected with HIV					
Fear over detection of disease or genetic predispositions					
Fear that the data generated from the research can result in stigmatization and discrimination					
Fear over the commercial use of the samples					
Fear that the government could have the access to the samples					
Fear that the insurance companies could have the access to the samples					
Fear that the employers could have the access to the samples					

12. Which information about the donor should be protected?

	Definitely yes	Rather yes	Rather no	Definitely no	I do not know
Address data					
The national identification / identity number					
Health condition/previous diseases					
About addictions					
About diseases in the family					
About genetic susceptibility to somatic diseases					
About genetic susceptibility to mental disorders					
About sex life					
About religion/confession					
About profession					
About earnings					
About nationality / ethnicity					
About political preferences					
All information should be protected					

13. Samples taken from donors for research purposes should be:

☐ Pseudonymized (reversibly coding, i.e. in case of detecting a disease)
☐ Anonymized (irreversibly coded, so that donor data cannot be determined)
☐ I do not know

14. When the donor wants to withdraw from the research their sample should be:

☐ Anonymized (irreversible coded) but available for further research
☐ Destroyed
☐ Prohibited from use in further research
☐ I do not know

15. What type of consent would you prefer while donating your samples to biobank?

☐ Blanket (open-ended permission without any limitations and the need to renewed consent)
☐ Specific consent (for one experiment with well-defined aim / before every *research* that involves my samples)
☐ Broad consent (general consent for a broad range of future studies but subjected to specified limitations and restrictions stated in the consent form)
☐ Tiered consent (individually selected categories of research or research uses e.g. specific diseases, i.e. cancer or neurological diseases, or research conducted only by specified institutions, i.e. publicly-funded but not private)
☐ Consent delegated to bioethical committee
☐ I do not know

16. When should the biobank ask donors for permission to use their samples?

	Definitely yes	Rather yes	Rather no	Definitely no	I do not know
Before every new research					
If a new research project differs from the original project					
If it is intended to be used by researchers outside the institution donors donated to					
If it is intended for use by foreign institutions					
Does not have to ask if the doner consent while donating					

17. While donation the samples the donors should rather

☐ Specify the types of research for which their specimens may be used
☐ Specify the types of research for which their specimens may not be used
☐ I do not know

18. Who should own the rights to the samples donated to the biobank?

- ☐ Biobank
- ☐ Donors
- ☐ Both biobank and donor
- ☐ I do not know

19. Who should profit from the biobank research?

- ☐ Sponsor of the research/Biobank owner
- ☐ Donors
- ☐ Both biobank and donor
- ☐ I do not know
